# Plasmid sequences and availability of a two-plasmid system for CRISPRi knockdown of *Clostridioides difficile* genes without antibiotic selection

**DOI:** 10.1128/mra.00116-25

**Published:** 2025-04-28

**Authors:** Michelle Chua, Daniel Erickson, James Collins

**Affiliations:** 1 Department of Microbiology & Immunology, University of Louisville 5170, Louisville, Kentucky, USA; 2 Center for Predictive Medicine, University of Louisville 5170, Louisville, Kentucky, USA; 3 Center for Microbiomics, Inflammation and Pathogenicity, University of Louisville 5170, Louisville, Kentucky, USA; Indiana University Bloomington, Bloomington, Indiana, USA

**Keywords:** CRISPRi, *Clostridioides difficile*, antibiotic-free maintenance, dCas9

## Abstract

A two-plasmid CRISPRi system for *Clostridioides difficile* that does not require antibiotic maintenance was developed. pJAK184.tetR.PT5-3.dCas9 contains an optimized tetracycline-inducible *dCas9* for chromosomal insertion. pJC.15A.sgRNA.TA encodes a toxin-antitoxin system for stable maintenance, and *mCherry*, which is exchangeable for a customized sgRNA. We demonstrate the knockdown of the essential gene *walA*.

## ANNOUNCEMENT

Tools for the genetic manipulation of *C. difficile* are essential for understanding its pathogenesis. Here, we describe a CRISPRi system that does not require antibiotic maintenance, enabling the study of essential genes both *in vitro* and *in vivo*.

**Fig 1:**
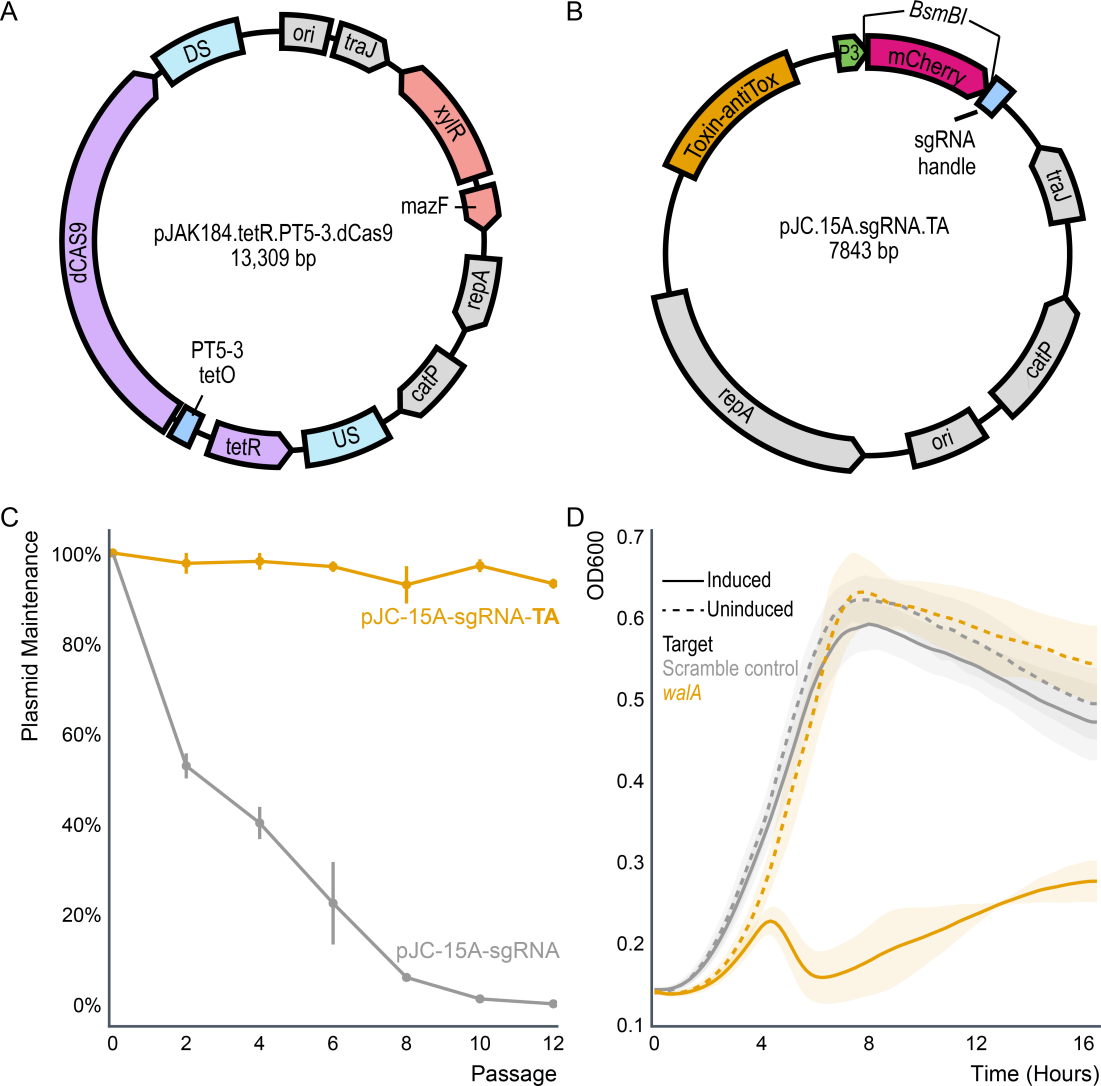
CRISPRi system. (A) Map of pJAK184.tetR.PT5-3.dCas9. This plasmid contains dCas9 driven by an optimized promoter (PT5-3) and was designed to be integrated into the *C. difficile* genome using mazF as the counter-selectable marker. (B) Map of pJC.15A.sgRNA.TA. This plasmid contains mCherry, which can be removed to integrate the sgRNA of interest. Once transformed into *C. difficile*, pJC.15A.sgRNA.TA remains stable due to the presence of a toxin-antitoxin system. (C) The introduction of a putative *C. difficile* toxin-antitoxin system allows for stable plasmid maintenance without the need for antibiotics. Three biological replicates of each strain were passaged every 12 h in BHIS medium without selection and subsequently plated onto BHIS agar with and without antibiotics. (D) Growth in the TY medium was inhibited when walA was targeted via CRISPRi. Strains were grown in a medium with or without anhydrotetracycline (50 ng/mL) to induce dCas9.

pJAK184.tetR.PT5-3.dCas9 contains catalytically inactive Cas9 (dCas9) under the control of an optimized tetracycline-inducible promoter (Fig. 1A). Homologous regions enable integration into the *C. difficile* chromosome downstream of the bacitracin permease gene, and a xylose-inducible mazF enables the selection of double crossovers, as described by Fuchs et al. (1). The plasmid pJC.15A.sgRNA.TA encodes mCherry flanked by *BsmBI* restriction sites directly upstream of the tracrRNA (Fig. 1B). The restriction sites enable the rapid replacement of mCherry with a CRISPR RNA target constructed by annealing oligo pairs with an overhang compatible with the backbone vector. To allow stable replication without the need for antibiotic selection, the plasmid contains a toxin-antitoxin (TA) system derived from a putative chromosomal TA system in *C. difficile* CD630, which is absent in the majority of *C. difficile* strains. The TA system enables stable plasmid maintenance without the need for antibiotics (Fig. 1C). This system replicates stably and can knock down essential genes without incurring fitness costs in the absence of induction (Fig. 1D).

The plasmids were constructed as follows: For Gibson assembly and *in vivo* recombination, DNA fragments were synthesized or amplified using primers containing 25 nucleotide homologous ends. The promoter sequence driving *dCas9* (PT5-3) was derived from Sorg et al. (2) and synthesized by Twist Bioscience. The xylose-inducible *mazF*, origin of replication, *repA*, and *catP* genes were amplified from pJAK184 using primers FFO-362 and FFO-363 (1). The bacitracin permease upstream and downstream arms and *tetR* and dCas9 genes were assembled via Gibson assembly from their respective sources, and the entire region was amplified using primers oMC396 and oMC397.

The mCherry gene was amplified from the pPEPZ-sgRNAclone plasmid using primers oJC842 and oJC714. Primers oJC850 and oJC845 were used to amplify the p15A ori gene from pGEN222. *C. difficile* replication and *traJ*/*catP* genes were amplified from pRPF185 using primers oJC846/oMC221 and oJC715/oJC851, respectively. Primers oMC207 and oMC220 were used to amplify the toxin-antitoxin system from *C. difficile* CD630 genomic DNA. Fragments were assembled via *in vivo* recombination (3). Strains, plasmids, and primers are listed in Table 1.

**TABLE 1:** List of strains, plasmids, and primers used in this study

Strains/plasmids/primers	Relevant features	Source
pJAK184.tetR.PT5-3.dCas9		This work
pJAK184	Xylose-inducible *mazF*	(1)
pRPF185	*tetR* gene	(4)
pIA33	dCas9 gene	(5)
pJC.15A.sgRNA.TA		This work
pPEPZ-sgRNAclone	mCherry gene	(6)
pGEN222	p15A ori	(7)
pRPF185	*C. difficile* replication genes, *traJ* gene, and *catP* gene	(4)
*C. difficile* CD630	Toxin-antitoxin system	
oMC396	AAATACGGTGTTTTTTGTTACCCTAGGGGAGAAGTAAAAGTTCCAGT	
oMC397	TTTGGTCATGAGATTATCAAAAAGGAGTCAACTCTATACTTTTATCCTTAT	
oJC842	AATAATCATTGATTGCTCGAGCGCTAAAACTATTAATCTTATC	
oJC714	CCAGGAGAGTTGTTGCGTGGGCTCGGAGATGTGTA	
oJC850	AGGCGGTAATACGGTTCGGCCTAGGAGATACTTAACA	
oJC845	TGCTAGATTCTAAAATAGCAAATTAAGCAGAAGGCCATCC	
oJC846	GCCTTCTGCTTAATTTGCTATTTTAGAATCTAGCATTTCCAATGCTTA	
oMC221	TATAAGTATTGAGGCGGCTGTGAAAGTGGGTCTTAAGGTACCATAAAAAT	
oJC715	ATCTCCGAGCCCACGCAACAACTCTCCTGGCGCAC	
oJC851	TATCTCCTAGGCCGAACCGTATTACCGCCTTTGAGT	
oMC207	CCCACTTTCACAGCCGCCTCAATACTTATAAAG	
oMC220	ATGATTATTCATTTTTTTTATATAAACAATGAAATTCAAG	

Following assembly, pJAK184.tetR.PT5-3.dCas9 was conjugated into *C. difficile* R20921 and chromosomally inserted downstream of the bacitracin permease gene. Clean insertions were confirmed by Sanger sequencing. The plasmid pJC.15A.sgRNA.TA was digested with *BsmBI,* sgRNAs targeting either *walA* or a scrambled control were inserted, and the plasmids were conjugated into dCas9 containing *C. difficile*. Although the negative (scramble) control grew like wild-type, regardless of induction, the R20291 strain with the *walA* sgRNA plasmid displayed significantly reduced growth when induced but grew normally when uninduced (Fig. 1D). Growth studies were conducted in the absence of antibiotics.

## Data Availability

Plasmids and complete sequences are available through Addgene depositories 232482 and 232483.
